# Diversity and comparative genomics of *Microviridae* in *Sphagnum-* dominated peatlands

**DOI:** 10.3389/fmicb.2015.00375

**Published:** 2015-04-28

**Authors:** Achim Quaiser, Alexis Dufresne, Flore Ballaud, Simon Roux, Yvan Zivanovic, Jonathan Colombet, Télesphore Sime-Ngando, André-Jean Francez

**Affiliations:** ^1^UMR CNRS 6553 – ECOBIO, Université de Rennes 1Rennes, France; ^2^Department of Ecology and Evolutionary Biology, University of Arizona, Tucson, AZUSA; ^3^CNRS, UMR 8621, Université Paris SudOrsay, France; ^4^CNRS, Laboratoire Microorganismes: Génome et Environnement – UMR 6023, Université Blaise PascalClermont-Ferrand, France

**Keywords:** virus ecology, viral metagenomics, virus diversity, ssDNA phage, *Microviridae*, *Gokushovirinae*, *Aravirinae*, *Stokavirinae*

## Abstract

*Microviridae*, a family of bacteria-infecting ssDNA viruses, is one of the still poorly characterized bacteriophage groups, even though it includes phage PhiX174, one of the main models in virology for genomic and capsid structure studies. Recent studies suggest that they are diverse and well represented in marine and freshwater virioplankton as well as in human microbiomes. However, their diversity, abundance, and ecological role are completely unknown in soil ecosystems. Here we present the comparative analysis of 17 completely assembled *Microviridae* genomes from 12 viromes of a *Sphagnum-*dominated peatland. Phylogenetic analysis of the conserved major capsid protein sequences revealed the affiliation to *Gokushovirinae* and *Pichovirinae* as well as to two newly defined subfamilies, the *Aravirinae* and *Stokavirinae*. Additionally, two new distinct prophages were identified in the genomes of *Parabacteroides merdae* and *Parabacteroides distasonis* representing a potential new subfamily of *Microviridae*. The differentiation of the subfamilies was confirmed by gene order and similarity analysis. Relative abundance analysis using the affiliation of the major capsid protein (VP1) revealed that *Gokushovirinae,* followed by *Aravirinae,* are the most abundant *Microviridae* in 11 out of 12 peat viromes. Sequences matching the *Gokushovirinae* and *Aravirinae* VP1 matching sequences, respectively, accounted for up to 4.19 and 0.65% of the total number of sequences in the corresponding virome, respectively. In this study we provide new genome information of *Microviridae* and pave the way toward quantitative estimations of *Microviridae* subfamilies.

## Introduction

Viruses, in particular bacteriophages, have been identified in several ecosystems, and represent the most abundant biological entities on Earth. In marine ecosystems and freshwater lakes they are present at high concentrations of about 10^7^ particles/mL on average (Wommack and Colwell, 2000). Viruses can control the microbial abundance and influence the composition of microbial communities by lysing their host organisms (Fuhrman and Schwalbach, 2003). In addition, they carry a highly diverse pool of genetic elements that might be exchanged with their hosts, thereby contributing to adaptation processes through the acquisition of new functions (Pedulla et al., 2003). Despite these potential influences the importance is almost solely illustrated in aquatic ecosystems (Wommack and Colwell, 2000; Suttle, 2007), and little is known about the diversity of viruses and in particular about ssDNA viruses in soil ecosystems. ssDNA viruses are divided into two families of bacteriophages (*Inoviridae* and *Microviridae*) and five eukaryotic viruses (King et al., 2012). Recent studies revealed that *Microviridae* seem to be ubiquitous. Through metagenomic approaches, *Microviridae* were identified in marine environments (Angly et al., 2006; Tucker et al., 2011; Labonté and Suttle, 2013a,b), freshwater habitats (López-Bueno et al., 2009; Roux et al., 2012a,b), human gut or feces (Roux et al., 2012b), stromatolites (Desnues et al., 2008), dragonflies (Rosario et al., 2012), sewage and sediments (Hopkins et al., 2014), and even as temperate phages integrated in the genomes of Bacteroidetes species (Krupovic and Forterre, 2011). These studies contribute significantly to the accumulation of *Microviridae* specific genomic information allowing more precise comparative genome analyses. These revealed that all *Microviridae* genomes possess several homologous genes including a well-conserved gene coding for the major capsid protein VP1, a roughly 500 amino acid long protein. This protein can be used as phylogenetic marker facilitating delineation of *Microviridae* clades or subfamilies (Wommack and Colwell, 2000; Desnues et al., 2008; Roux et al., 2012b; Labonté and Suttle, 2013b; Hopkins et al., 2014). All these studies applied whole-genome amplification methodology that preferentially amplifies small circular DNA templates such as ssDNA bacteriophages, which consequently introduces biases (Fuhrman and Schwalbach, 2003; Kim and Bae, 2011). We took advantage of this bias in that the ssDNA bacteriophages were likely preferentially enriched allowing for more targeted diversity analyses and exploration of new *Microviridae* genomes.

In this study, we analyzed 18 complete *Microviridae* genomes reconstructed through *de novo* assembly of reads from 12 viromes one microbial metagenome and obtained from peat soil and water samples collected in a *Sphagnum-*dominated peatland. Detailed phylogenetic analysis and genome comparisons revealed two new subfamilies. Combined with the available genomic information of *Microviridae* we provide new insights into the diversity, distribution and abundance of the *Microviridae* subfamilies.

## Materials and Methods

### Sampling and Accession to 12 Peat Viromes

Twelve samples were recovered from a *Sphagnum-*dominated peatland at “les Pradeaux mire” in the French Massif Central (3°55E; 45°32N) at an altitude of 1,250 m. Water and peat soils were both sampled in young states of peatland dynamics, called ‘Fen’ with *Sphagnum fallax* and *Carex rostrata* vegetation, and in older states of dynamics, called ‘Bog’ with *S. magellanicum* and *S. capillifolium, Andromeda polyfolia,* and *Eriophorum vaginatum* (Francez and Vasander, 1995). One peat sample (-5 cm to -15 cm depth) for each Fen and Bog was collected in June, August and October 2011 as well as three biological replicates of water that was extracted from *Sphagnum*-peat and filtered at 125 μm from both Fen and Bog (March 2012). Viruses were concentrated using PEGylation (Wommack and Colwell, 2000; Colombet et al., 2007; Suttle, 2007). Extracellular DNA was digested with DNAse RQ1 (Promega) at 37°C for 1 h. Viral DNA was extracted with the Nucleospin Extract II kit (Macherey–Nagel). Whole genome amplification (WGA) reactions were run in triplicate for each sample using Genomi-Phi following the instructions of the manufacturer (GE Healthcare) and subsequently pooled. Library construction and pyrosequencing was performed at the “Functional and Environmental Genomics” platform (OSUR, Rennes, France). After sequence quality and size trimming we obtained 481,402, and 618,487 reads from Fen and Bog, respectively, with an average length of 415 bp. *Microviridae* genomes reported in this publication have been deposited in the Sequence Read Archive under the study accession number KM589498–KM589516.

### Assembly, Annotation, and Comparative Genome Analysis

The peat viromes were assembled separately into contigs using the meta-assembler of CAMERA under standard conditions (Seshadri et al., 2007; King et al., 2012). The circularity of the genomes was checked manually. Only *Microviridae* genomes that showed overlapping reads at the start and end of the contigs, confirming their circular genomes were retained. Sequence analysis was performed using an integrated WEB-based annotation platform adapted to metagenomic sequence analysis (Genomic toolbox; Angly et al., 2006; Tucker et al., 2011; Labonté and Suttle, 2013a,b; Zivanovic et al., 2014; http://www-archbac.u-psud.fr/projects/aq_virome/aq_virome.html). Candidate coding sequences (CDSs) were defined with PRODIGAL (version 2.60; López-Bueno et al., 2009; Hyatt et al., 2010; Roux et al., 2012a,b). Semi-automated annotation was performed to identify genes by sequence similarity and coding probability using BLASTP (Altschul et al., 1997; Roux et al., 2012b) against the RefSeq NR protein database (GenBank), SWISSPROT (version 57.11), and COG databases (COG + KOG, seven eukaryotic genomes). Manual annotation was completed within the above-mentioned platform. Gene order comparison was performed using the platform tool GENOMAPPER (Desnues et al., 2008; Zivanovic et al., 2014) combined with multiple alignment and phylogenetic analysis. To allow the interactive visualization of genomic fragment comparisons, we used Artemis Comparison Tool ACTv.6 (Carver et al., 2012; Rosario et al., 2012).

The presence of *Microviridae* in peat metagenomes that were constructed from corresponding peat samples without virus enrichment (Quaiser et al., unpublished data) was analyzed by BLASTN and reads matching the virome-assembled *Microviridae* genomes were selected. The *Microviridae* contig from the metagenome was assembled manually using 21 reads. Since the reads showed nearly 100% identity to the *Gokushovirinae* genome Fen7875_21, it was used as a reference for recruitment of metagenome reads.

### Phylogenetic Analysis

Sequences of full length major capsid protein and replication protein as well as the complete nucleic acid sequences of the *Gokushovirinae* genomes were aligned using MUSCLE (Edgar, 2004; Hopkins et al., 2014). Alignments were manually edited using ARB (Ludwig et al., 2004; Krupovic and Forterre, 2011). Gaps and ambiguously aligned positions were excluded from phylogenetic analysis. Maximum likelihood trees were reconstructed using TREEFINDER (Jobb et al., 2004) applying a JTT model (amino acid) and GTR3 (nucleic acid) of sequence evolution with a four category discrete approximation of a distribution plus invariant sites. The best-fit models were determined using TREEFINDER. Maximum likelihood bootstrap proportions were inferred using 1000 replicates.

### Major Capsid Protein Structural Modeling

I-TASSER (Roy et al., 2010) was used to assess a first model of a VP1 protein from each of the two new clades (Bog5275_51 for *Aravirinae*, Fen51_42 for *Stokavirinae*). These initial models were processed through multiple steps of loops refinement with MODELER (Eswar et al., 2008) to improve their quality (PROSA-WEB; Wiederstein and Sippl, 2007). The final quality *Z*-scores of the obtained models was -5.15 and -4.59 for Bog5275_51 (*Aravirinae*) and Fen51_42 (*Stokavirinae*), respectively, which according to the ProSA-web server is in the range of X-ray based models, though slightly higher than the two reference VP1 models (-6.4 for φX174 F and -6.14 for SpV4 VP1). Visualization of the structural models and sequence conservation was performed with UCSF CHIMERA (Pettersen et al., 2004).

### Relative Abundance of *Microviridae*

**FIGURE 1:**
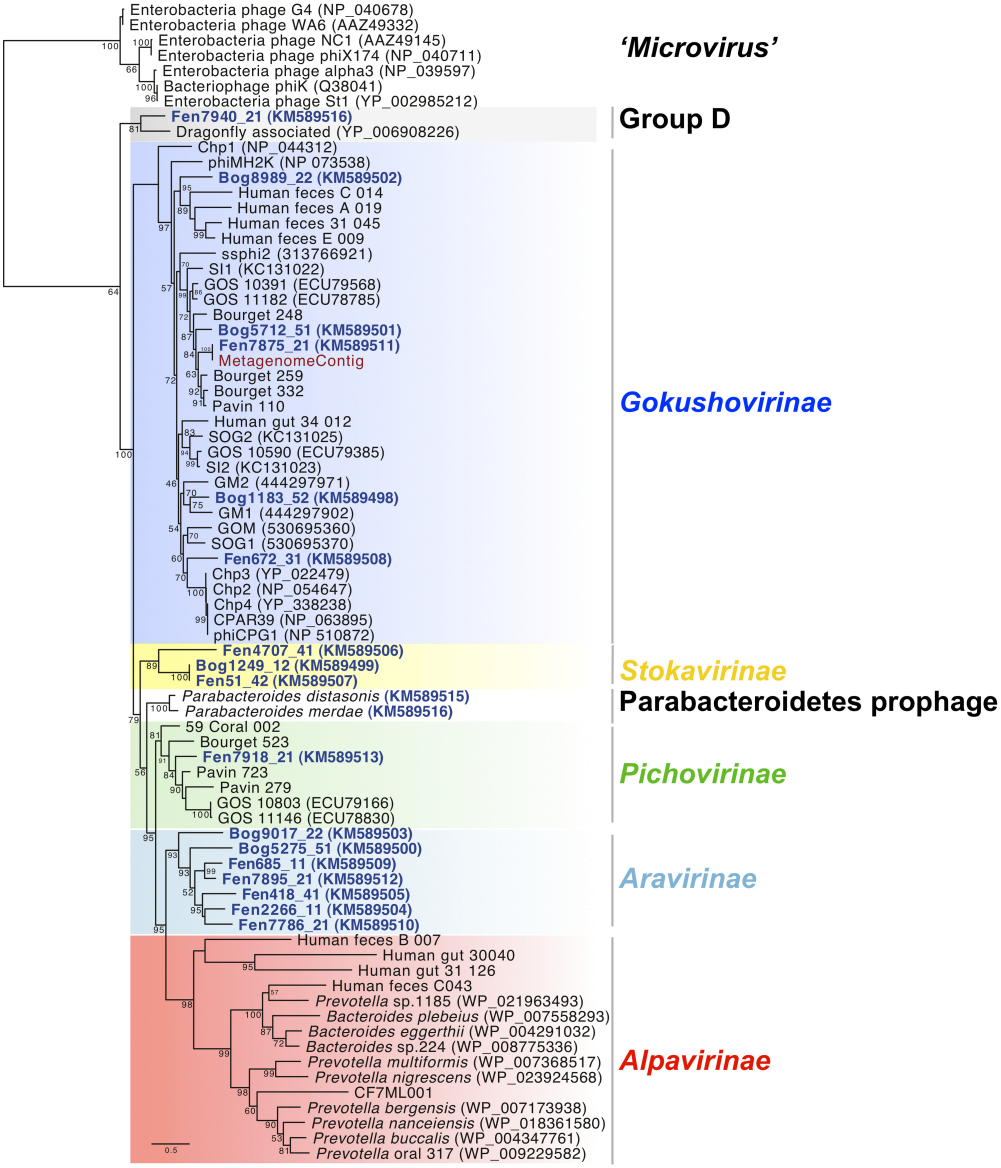
**Maximum likelihood phylogenetic analysis of full-length major capsid protein sequences present in the *Microviridae* genomes from *Sphagnum*-dominated peat viromes.** A total of 253 unambiguously aligned positions from 76 sequences were used in the analysis. Bootstrap values are indicated at the nodes. The scale bar indicates the number of substitutions per position for a unit branch length. Chlamydiaphages: Chp 1–4, CPAR39, phiCPG1; Bdellovibriophage: phiMH2K.

We compiled available full-length major capsid protein sequences from the complete *Microviridae* genomes to a total of 88 sequences and attributed the taxonomic affiliation according to the phylogenetic analysis in **Figure 1**. BLASTX analysis against the major capsid protein sequences was performed for the 12 *Sphagnum*-peat viromes and 69 viromes from public databases. To assure the reliability of matches a strict cut-off value of 1e^-10^ was applied. The number of matches was normalized to the total number of reads in the different viromes.

## Results and Discussion

### Assembly, Identification, and Analysis of Complete *Microviridae*-Like Genomes

To get insights into the genome structure and the distribution of *Microviridae*, reads from 12 peat viromes were assembled separately into contigs using the meta-assembler of CAMERA (Seshadri et al., 2007). In total 840 contigs longer than 3 kbp were obtained. Of these, 107 contigs showed high sequence similarity to the major capsid protein VP1 of *Microviridae* identified with TBLASTX analysis and by GENE RELATIONS and SYNTENY ANALYSIS tools from the Genomics toolbox (see Material and Methods). Only contigs corresponding to complete circular genomes were retained. In total, we obtained 17 new complete bacteriophage genomes affiliated to *Microviridae*. Sequencing coverage ranged from 8.31 to 94.63 times (Supplementary Table S1). One additional genome was assembled from reads of a microbial metagenome constructed from a peat sample without viral enrichment (Quaiser, unpublished data). Interestingly, several genomes assembled independently from different samples exhibited very high levels of similarity, thus validating the assembly process of viral genomes from metagenomic reads used in this study and confirming that the obtained assemblies were not chimeric and represented real *Microviridae* genomes.

### Diversity of Peat *Microviridae* Accessed by Phylogenetic Analysis of the Conserved Major Capsid Protein VP1

To assess the diversity of the new *Microviridae*-affiliated genomes, phylogenetic analysis was performed using the translated sequence of the gene coding for the major capsid protein VP1 (**Figure 1**). Eight different clades were formed using representative sequences from already identified *Microviridae* subfamilies and VP1 sequences from the 18 peat *Microviridae* genomes.

Five *Microviridae* genomes recovered from the viral fraction of the peat samples as well as the genome assembled from a peat metagenome clustered within the subfamily of *Gokushovirinae*. Two of these genomes derived from Fen and three from Bog samples. Peat *Gokushovirinae* were clearly separated from cultured ssDNA bacteriophage representatives (Chlamydiaphages: Chp 1–4, phiCPG1, CPAR39; Bdellovibriophage: phiMJ2K) and clustered with recently assembled *Gokushovirinae* genomes from planktonic microbial communities (GM1; Labonté and Suttle, 2013a), freshwater lake (Bourget_248, Bourget_259; Roux et al., 2012a), and human feces (Roux et al., 2012b).

One new clade was formed by three genomes from three different viromes (Fen4707_41, Bog1249_12, Fen51_42) representing a putative novel subfamily. We propose to name it *Stokavirinae* (Stoka: small in Sanskrit). A second new clade, which we propose to name *Aravirinae* (Ara: little in Sanskrit), consisted of seven new genomes assembled from five different peat viromes (Bog9017_22, Bog5275_51, Fen685_11, Fen7895_21, Fen418_41, Fen2266_11, Fen7786_21). Only one genome, Fen7918_21, was affiliated with the recently defined subfamily of *Pichovirinae* that, to date, consists of seven assembled genomes isolated from freshwater and marine environments (Roux et al., 2012b). Genome Fen7940_21 was affiliated with the dragonfly associated microvirus representing the second member of this clade of ssDNA microviruses (Rosario et al., 2012) and thereby confirming its distinction into another potentially novel subfamily (Group D). *Alpavirinae* that were identified as integrated prophages in Bacteroidetes genomes associated with human gut and oral microbiota (Krupovic and Forterre, 2011; Roux et al., 2012b) as well as sequences affiliated to the genus Microvirus (proposed subfamily *Microvirinae*) could not be identified in any of the peat viral genomes. Phylogenetic analysis of VP1 including recently amplified sequences from *Gokushovirinae* changed slightly the tree topology (Supplementary Figure S1; Labonté and Suttle, 2013a). In this case Bog9017_22 was clearly separated from *Aravirinae* and Fen7940_21 was apart from dragonfly associated microvirus. This is probably due to the shorter alignment imposed by the PCR-generated sequences that lower the resolution of the phylogenetic analysis.

Taxonomic affiliation was additionally determined using sequences of the replication protein (VP4), which is shared by all the *Microviridae* genomes (Supplementary Figure S2). While the replication protein is shorter and less conserved than the major capsid protein, phylogenetic analysis revealed similar clades with the sole exception of Bog9017_22, which moved from *Aravirinae* (major capsid protein phylogeny) to *Pichovirinae* in replication protein phylogeny. While this could hint for a chimeric assembly of the genome, it is noteworthy that the replication protein sequence is shorter and more variable, and therefore provides a less reliable phylogenetic signal than the VP1 sequence (Supplementary Figure S2; Roux et al., 2012b).

### Structure of the *Aravirinae* and *Stokavirinae* Major Capsid Protein

**FIGURE 2:**
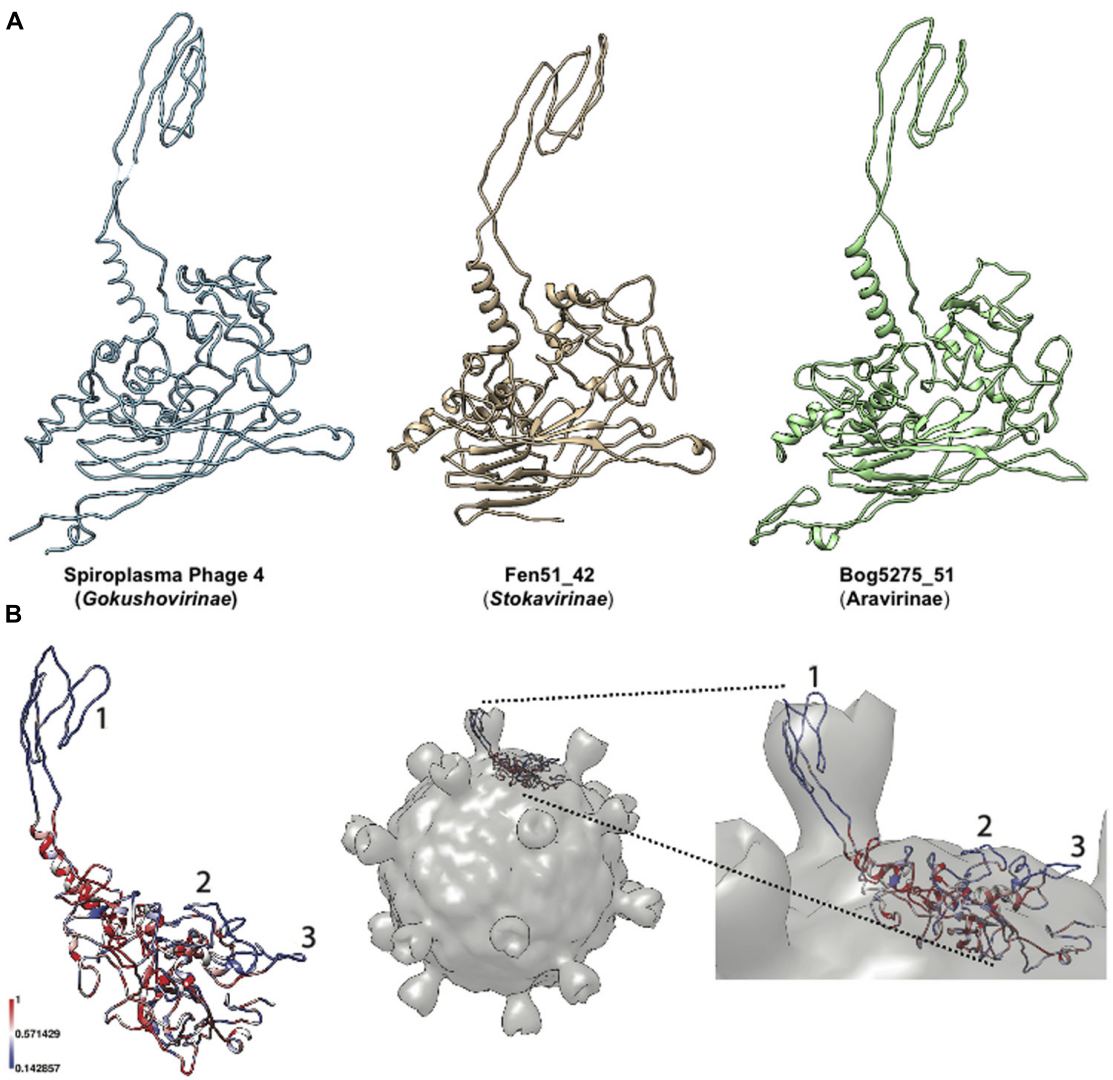
**Modeling of major capsid proteins. (A)** Three-dimensional models of *Microviridae* major capsid protein. Different subfamilies are depicted with, from left to right, the reference model from *Spiroplasma* Phage 4 (Pdb Id: 1KVP), representatives from *Stokavirinae* and *Aravirinae*. **(B)** Hypervariable regions of *Aravirinae* major capsid protein. The sequence conservation across the seven *Aravirinae* sequences was mapped on the three-dimensional model obtained for the Bog5275_51 genome. The three hypervariable regions were numbered (left panel). This model was mapped on the capsid structure of *Spiroplasma* phage 4 to estimate the position of these hypervariable regions relative to the whole virion (right panel).

Structural modeling of the major capsid protein from the two new *Microviridae* subfamilies indicated that they harbor a conserved eight-stranded β-barrel core (“viral jelly- roll”) and a loop extension (**Figure 2A**) similar to *Alpavirinae* and *Pichovirinae* which is known to form mushroom-like protrusions in SpV4 (Roux et al., 2012b). These protrusions, found in every type of *Microviridae* except for the *Microvirinae*, are thought to bind to host receptors (Roux et al., 2012b). Based on the seven different *Aravirinae*, we analyzed the level of residue conservation along the major capsid protein sequences. As expected, the protrusion loop is highly variable (region 1 on **Figure 2B**). Moreover we identified two additional variable regions backing the separation into a new subfamily (**Figure 2B**). Interestingly, when mapping the protein model on the virion structure, these two additional variable regions appear to be situated on the virion surface. We speculate that these regions may form additional outer structures involved in protein–protein interaction.

### Identification of *Microviridae* Prophages in Bacterial Genomes

**FIGURE 3:**
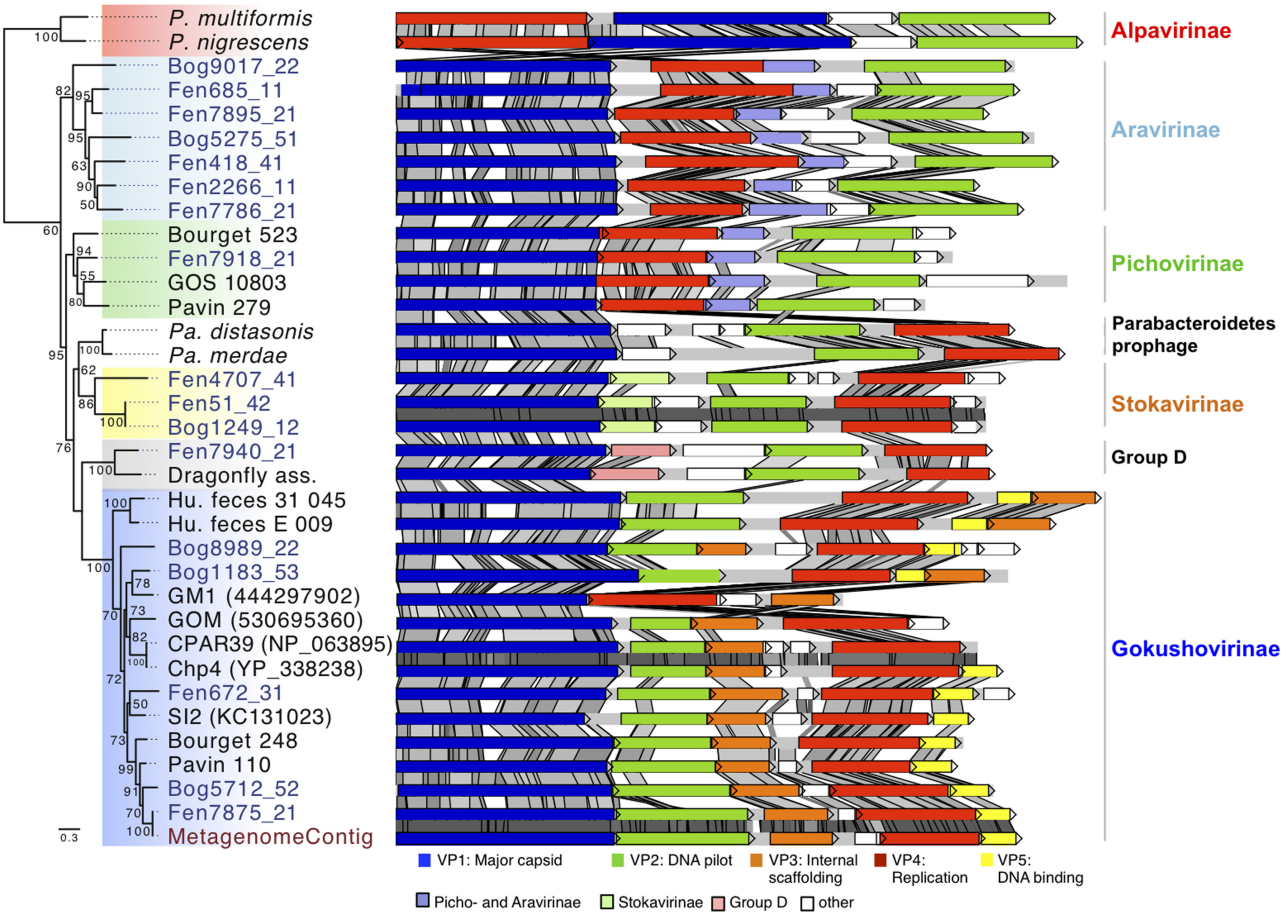
**Major capsid protein phylogeny and genome structure of major subfamilies of *Microviridae* bacteriophages.** The affiliation of the peat genomes to *Microviridae* is shown by major capsid protein phylogeny (Maximum likelihood, 252aa positions, 1000 iterations, JTT+G model). Bootstrap values above 50% are indicated at the nodes. The genome structures of the assembled viral peat genomes (blue) were compared to other *Microviridae* genomes. Pairwise comparison (TBLASTX) was visualized with ACT (Carver et al., 2005). Gray shading indicates the level of similarities. Homologous genes specific for some subfamilies are color-coded as indicated in the legend. *P. multiformis, Prevotella multiformis* (WP_007368517)*; P. nigrescens, Prevotella nigrescens* (WP_023924568)*; Pa. distasonis, Parabacteroides distasonis* (ZP_17317332)*; Pa. merdae, Parabacteroides merdae* (WP_022322420). Dragonfly, Dragonfly associated phage (YP_006908226); GOS_10803 (ECU79166); GM1 (444297902); GOM (530695360); CPAR39 (NP_063895); Chp4 (YP_338238); SI2 (KC131023); Metagenome: genome assembled from the metagenome.

Blast searches with the major capsid protein sequences from the assembled genomes showed similarities to VP1 proteins encoded in the genomes of the bacteria *Parabacteroides distasonis* and *Parabacteroides merdae* (Sakamoto and Benno, 2006). A detailed inspection of the genomic environment of the *Parabacteroidetes* VP1 gene showed that genes coding for homologs of VP2 and VP4 were located next to the VP1 gene in a 5 kbp region. This indicates the presence of a prophage affiliated to *Microviridae* in both *Parabacteroidetes* species. In order to compare their genomic organization, these identified prophage regions were cut from the two genomes and considered as circular genomes. For comparative analysis the genome start was arbitrarily fixed at VP1. Synteny analysis confirmed that the two prophages shared a common ancestor showing the same gene order (VP1-ORF1-ORF2-ORF3-VP2-VP4 for *Parabacteroides distasonis* and VP1-ORF1-VP2-VP4 for *Parabacteroides merdae*; **Figure 3**). The gene coding for an uncharacterized protein (ORF1) located downstream of the VP1 coding gene was specific to the two prophages and did not match with genes from other *Microviridae* genomes or from the NCBI non-redundant database. This strengthens the hypothesis that these prophages represent a distinct subfamily of *Microviridae* as suggested by the phylogenetic analysis of the major capsid protein sequences (**Figure 1**).

### Comparative Genome Analysis

To get further insights into the diversity and evolution of the new *Microviridae* genomes the genome structures and gene sequence conservation levels were compared (**Figure 3**). The characteristic genes encoding the major capsid protein (VP1), replication protein (VP4), and DNA pilot protein (VP2) were identified in all *Microviridae* genomes. All analyzed *Gokushovirinae* possessed one additional gene coding for an internal scaffolding protein (VP3) and in most cases a DNA binding protein (VP5). Both genes were absent in all other subfamilies of *Microviridae*. The sequence similarity of VP3 and VP5 among the peat *Gokushovirinae* ranged from 31 to 100 and 26 to 100% identity, respectively. The gene order in *Gokushovirinae* was relatively well conserved, but we could distinguish two clades. In one clade the gene order consists of VP1, VP2, VP3, VP4, and VP5 (i.e., Fen672_31) while in the other clade the organization was VP1, VP2, VP4, VP5, and VP3 (Bog1183_53, Human_feces_E_009, and Human_feces_31_045). However, this distinction was not supported by phylogenetic analysis of the major capsid protein or the replication protein.

The two genomes, representative for the Group D (Dragonfly associated and Fen7940_21), contain five genes. Compared to other groups, they possess two additional genes organized in the same order between VP1 and VP2. The gene situated downstream of VP1 was conserved with up to 50% similarity but only over 41% of their amino acid sequences (**Figure 3**, Group D, pink). Since no other matches to this gene were found, neither in the NCBI non-redundant protein database nor in other peat *Microviridae* genomes, this gene was a unique feature for this clade confirming the distinction from other *Microviridae* clades. The second protein encoded upstream of VP2 appeared to be unique to each genome as they exhibited no similarity between each other or with proteins found in the NCBI nr database.

The three *Stokavirinae* genomes showed a similar organization. The protein encoded downstream of the major capsid protein gene was homologous within the *Stokavirinae* (**Figure 3**, light green) but without similarity to *Parabacteroidetes* prophage proteins or to the NCBI NR database. *Stokavirinae* possess a characteristic additional ORF downstream of VP4 that is absent in other subfamilies confirming that they present a distinct clade. Interestingly, the two genomes Bog1249_12 and Fen51_42 showed 99% nucleic acid identity while the third Fen4704_41 was more distant. Both were assembled independently not only from different viromes but also from bog and fen samples recovered at different dates. This indicates that the viral community was at least partially shared among different peat samples.

### Whole Genome Phylogeny and Comparative Genome Analysis of *Gokushovirinae*

**FIGURE 4:**
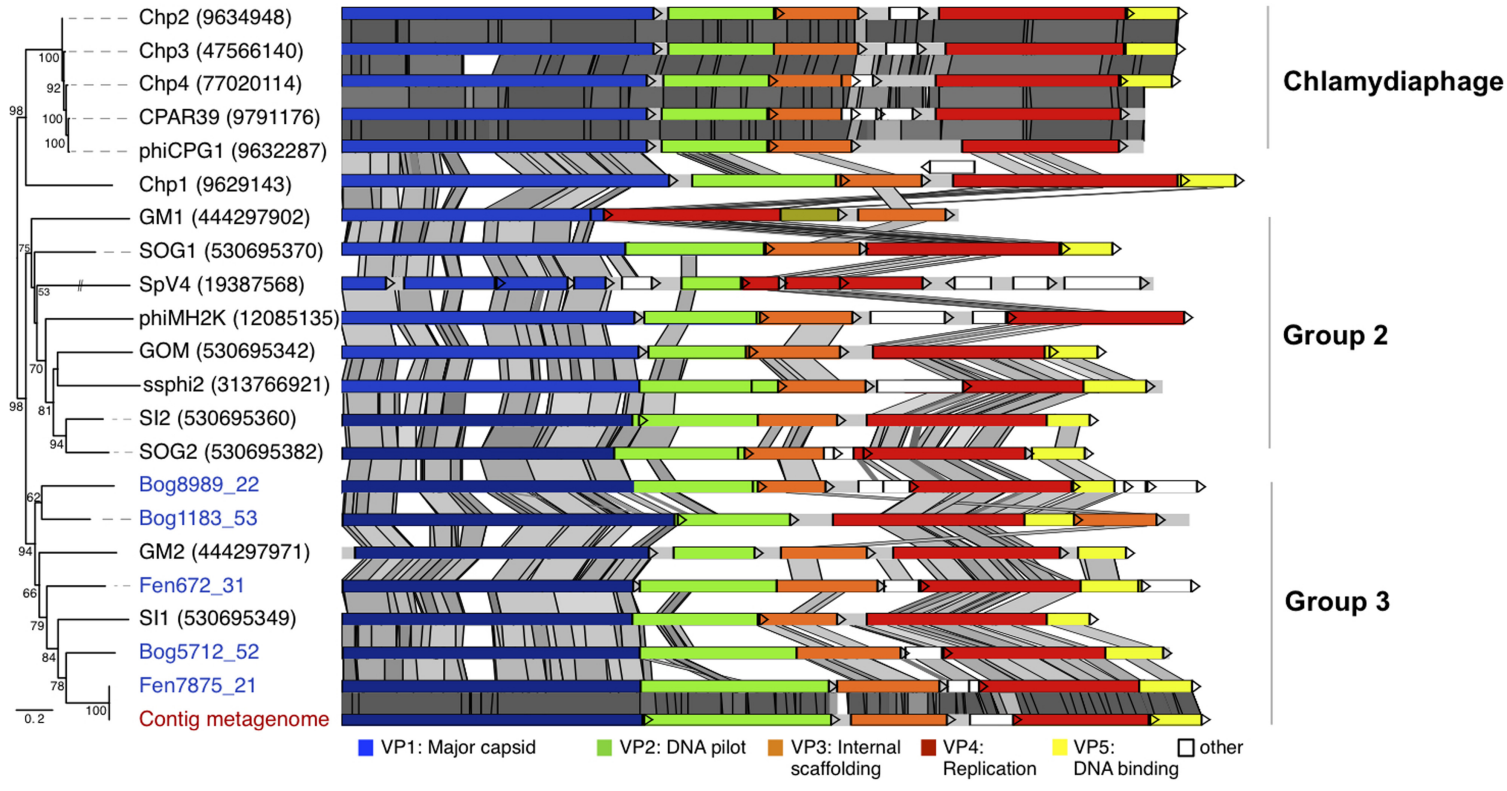
**Whole genome phylogeny and conserved genome structure of peat *Gokushovirinae*.** The affiliation of the peat genomes to *Gokushovirinae* was shown by whole genome phylogeny (Maximum likelihood, 2052 positions, 1000 iterations, GTR3 model). Maximum likelihood bootstrap values above 50% are indicated at the nodes. The genome structure of the assembled viral peat genomes (virome blue, metagenome red) was compared to *Gokushovirinae*. Pairwise comparisons (TBLASTX) were visualized by ACT (Carver et al., 2005). Gray shading indicates the level of similarities.

In order to determine the precise affiliation of the *Gokushovirinae* peat genomes, whole genome phylogeny was performed for this subfamily (**Figure 4**). The phylogenetic tree obtained reveals three groups that could not be identified based on analysis of the sole VP1 sequences: *Chlamydiaphage Gokushovirinae*, Group 2 and Group 3. As reported recently (Labonté and Suttle, 2013a) all cultured *Gokushovirinae* deriving from *Chlamydia* species, with the exception of Chp1 that appeared more distantly related, clustered together and their genome sequences were strongly conserved (Chp2, Chp3, Chp4, CPAR39, and phiCPG1). Group 2 contained phiMH2K (*Bdellovibrio*) and SpV4 (*Spiroplasma*) as well as recently assembled genomes from marine seawater (SOG1, GOM, ssphi2, SOG2, SI2; Labonté and Suttle, 2013a) and marine sediments (GM1; Yoshida et al., 2013). Group 3 contained the five peat *Gokushovirinae* and two assembled marine genomes SI1 (Labonté and Suttle, 2013a) and GM2 (Yoshida et al., 2013). Four peat *Gokushovirinae* exhibited the typical gene ordering, but in Bog1183_53 the VP3 gene was found downstream of the gene encoding VP5. Identities among the analyzed genomes ranged from 44.1 to 100% identical positions (Supplementary Table S2). Interestingly, the metagenome derived genome showed 100% similarity to the Fen7875_21 genome.

### Relative Abundance of *Microviridae* Subfamilies in Peatland and Other Habitats Based on Matches to the Major Capsid Protein Sequences

**FIGURE 5:**
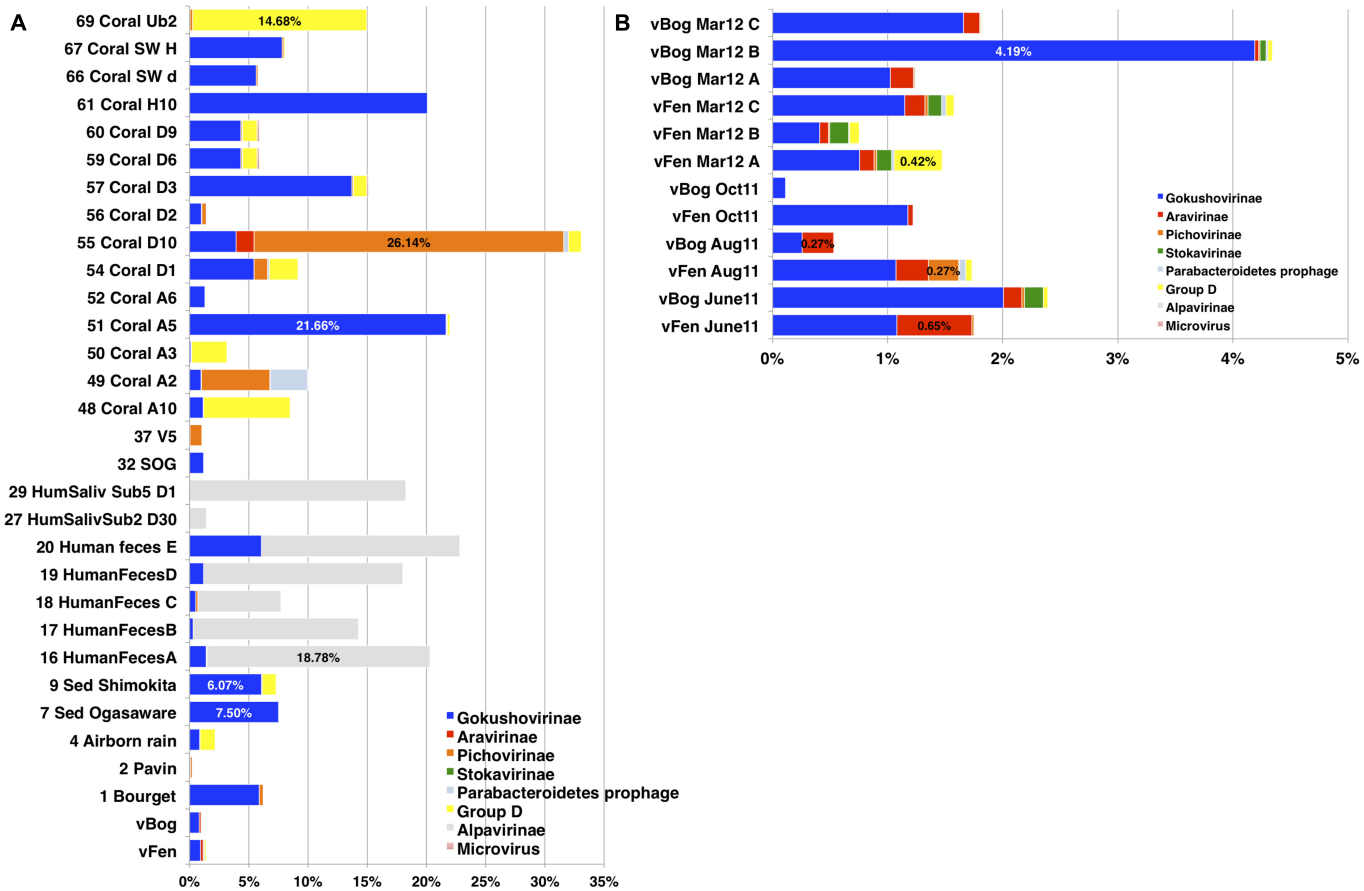
**Relative proportions of *Microviridae* subfamilies based on their content of major capsid protein sequences. (A)** VP1 match counts in 69 viromes from public databases; **(B)** in the 12 peat viromes. Matches to major capsid proteins were normalized to the number of total sequences in the viromes. Best matches were determined by BLASTX with an *e*-value 10^-10^ cut off.

In an attempt to estimate the distribution and abundance of *Microviridae* subfamilies the relative abundance of the major capsid protein coding genes was determined in the 12 peat viromes and in 69 additional viromes from 12 types of ecosystems (**Figure 5**). Quantitative estimations of viruses based on the detection of particular signature sequences is currently the best representative proxy, but should be considered with a grain of salt as WGA leads to overrepresentation of small ssDNA viruses (Kim and Bae, 2011), Nevertheless, to date WGA is the best way to recover sufficient viral genomic DNA for sequencing. Accordingly, all published viromes and the viromes included in this analysis were generated using this technique. Based on the phylogenetic analyses a database containing 88 major capsid protein sequences representing the different subfamilies of *Microviridae* was constructed. Each virome was searched with BLASTX against the major capsid protein sequence database and best matches were counted using strict count conditions (*e*-value 10^-10^). To take into account virome size variation, relative proportions of *Microviridae* subfamilies were obtained through the normalization of the matches by the total number of sequences in each virome. Taxonomic affiliation was determined according to the eight different subfamilies established in phylogenetic analyses (**Figure 1**). *Alpavirinae* represented the major subfamily of *Microviridae* in all human feces and human saliva viromes reaching up to 18.78% in the virome Human feces A (Kim et al., 2011; **Figure 5A**) while they were absent in all peat viromes as well as in most other analyzed viromes. *Gokushovirinae* were the most abundant subfamily in most peat viromes ranging from 0.11% in sample vBog_Oct11 to 4.19% in sample vBog_Mar12_B (**Figure 5B**). *Gokushovirinae* also represented the majority of the *Microviridae* retrieved in Shimokita and Ogasaware marine sediment viromes (Yoshida et al., 2013) representing 6.00 and 7.47%, respectively, and even reaching 21.66% of the Coral A5 virome (Soffer et al., 2014; **Figure 5A**).

With major capsid protein matches accounting for 0.27% of the total virome sequences, *Aravirinae* were the most abundant *Microviridae* subfamily in vBog_Aug11. They represented the second most abundant subfamily in vFen_June11, vFen_Aug11, vFen_Mar12_C, vBog_Mar12_A, and vBog_Mar12_B. *Stokavirinae* were the second most abundant in vBog_June11, vFen_Mar12_B, and vBog_Mar12_B. *Stokavirinae* and *Aravirinae* were not restricted to peatland. They were present in other viromes but seemed to be much less frequent as only 10 and 270 matches were detected, respectively, from a total of 282,508 major capsid protein matches identified in the 69 non-peat viromes. Matches to *Parabacteroidetes* prophages were low, ranging from 0 in vBog_June11 to 59 in vFen_Aug11, which corresponds to 0.055% of total viral sequences. Group D was the second most abundant subfamily in the virome vFen_Mars12_A with major capsid protein sequences accounting for 0.42%. As for other viromes, Group D affiliated major capsid protein sequences represented up to 14.68% in the virome Coral Ub2 (Soffer et al., 2014). *Pichovirinae* were present in all peat viromes with the exception of vBog Oct11 reaching up to 0.27% in vFen_Aug11. In the virome Lake Bourget (Roux et al., 2012b), where *Pichovirinae* were first detected, they accounted for 0.31%. The highest proportion was found in Coral D10 (Soffer et al., 2014), where the major capsid protein sequences of *Pichovirinae* accounted for 26.14% (**Figure 5A**) indicating that the viral community at the time of sampling consisted in the large majority of *Pichovirinae*.

The relative abundances of *Microviridae* subfamilies based on the analysis of the available viromes showed considerable variations, even between viral communities sampled from the same environment. For now, it is only possible to identify broad trends in the habitat preferences of *Microviridae* subfamilies. Further data and the identification of the corresponding hosts should help to determine the ecological and functional significance of the genome variations between *Microviridae* subfamilies.

## Conclusion

While bacteriophages are already considered as important biological actors in seawater, freshwater, and human gut ecosystems, this study sheds light onto the specific diversity of *Microviridae* in peatland. Namely, we identified two completely new *Microviridae* subfamilies from *Sphagnum*-peat habitats that are distinct by phylogenetic analysis and by their genome structure. By combining whole genome phylogeny, major capsid protein sequence phylogeny and genome structure analysis; we gained new detailed insights into the evolution and genomic diversity of *Microviridae* subfamilies. Notably, we highlighted new hypervariable regions in the major capsid protein sequence as well as the co-existence of different genome organizations. Diversity and relative abundance analysis using the major capsid protein as a marker gene revealed that *Gokushovirinae* have the largest environmental distribution and contributed the most to the pool of *Microviridae*-affiliated reads in a majority of viromes. Moreover, these new genomes allowed for the detection of a second group of *Microviridae* prophages, along with the *Alpavirinae* (Krupovic and Forterre, 2011), in two genomes of Bacteroidetes species that likely represent a novel *Microviridae* subfamily. These new *Microviridae* genomes significantly augment the currently available genome information facilitating subsequent comparative and diversity analyses.

## Conflict of Interest Statement

The authors declare that the research was conducted in the absence of any commercial or financial relationships that could be construed as a potential conflict of interest.
