# Supplementary material for: Diversity and comparative genomics of Microviridae in Sphagnum- dominated peatlands
Source: Front Microbiol. 2015 Apr 28;6:375. doi: 10.3389/fmicb.2015.00375 (PMC4412055; doi:10.3389/fmicb.2015.00375)
Supplement: Supplementary file 1 [file Presentation_1.PDF]

Supplementary Material

Diversity, distribution and comparative genomics of *Microviridae* in *Sphagnum*-peat soils

Achim Quaiser[1]*, Alexis Dufresne[1], Flore Ballaud[1], Simon Roux[2], Yvan Zivanovic[3], Jonathan Colombet[4], Télesphore Sime-Ngando[4] and André-Jean Francez[1]

[1]Université de Rennes 1, CNRS UMR6553 EcoBio, Rennes, France; [2]Department of Ecology and Evolutionary Biology, University of Arizona, Tucson, AZ, USA; [3]Université Paris Sud, CNRS UMR8621, 91405 Orsay, France. [4]Université Blaise Pascal, UMR CNRS 6023 LMGE, Clermont-Ferrand, France

***Correspondence:** Achim Quaiser, Université de Rennes 1, CNRS UMR6553 EcoBio, Rennes, France; achim.quaiser@univ-rennes1.fr

Supplementary Figure S1

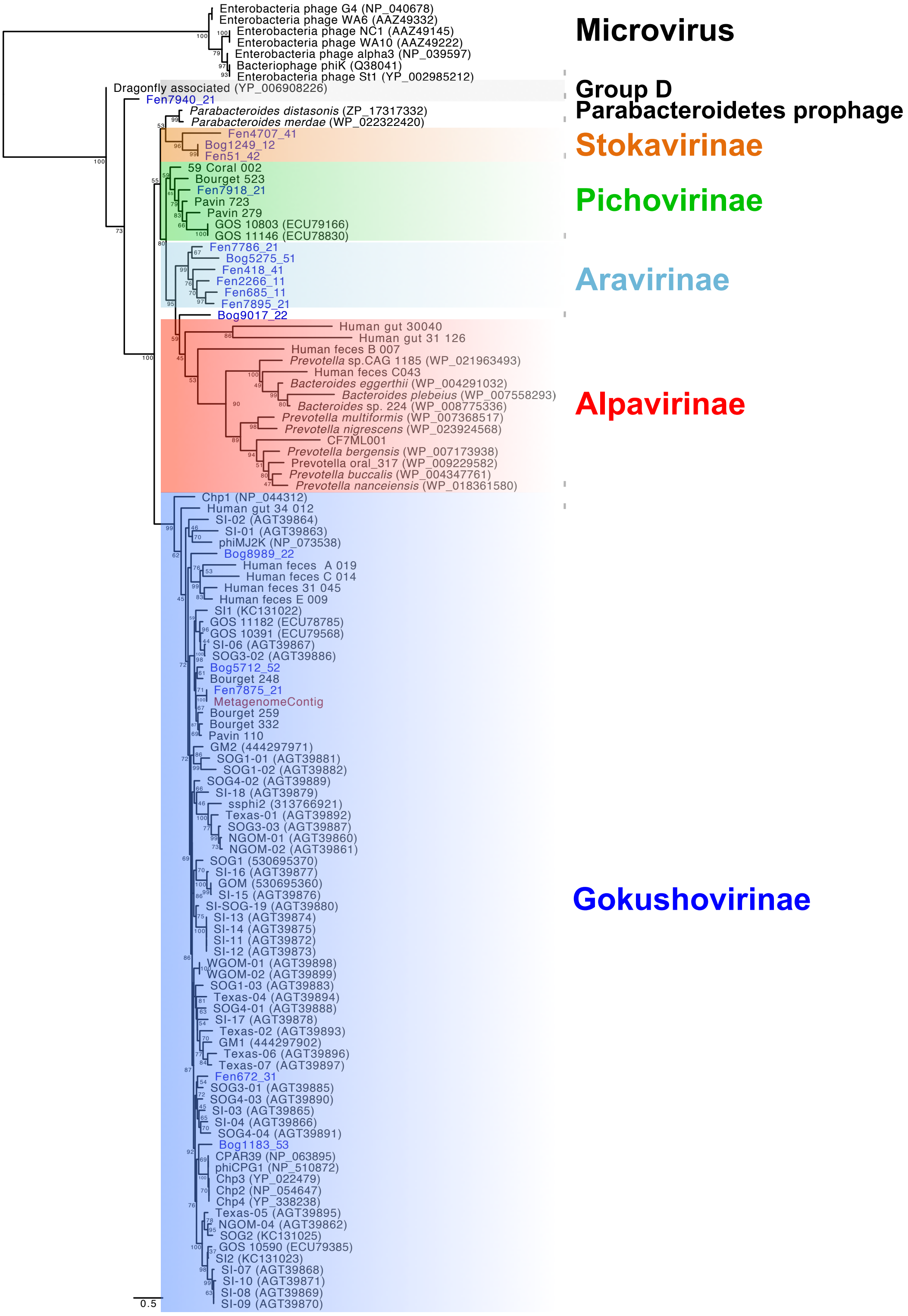

Supplementary Figure S1 Maximum likelihood phylogenetic analysis of major capsid protein sequences from *Microviridae* including recently amplified sequences. A total of 157 unambiguously aligned positions from 115 sequences were used in the analysis. Bootstrap values above are indicated at the nodes. The scale bar indicates the number of substitutions per position for a unit branch length.

Supplementary Figure S2

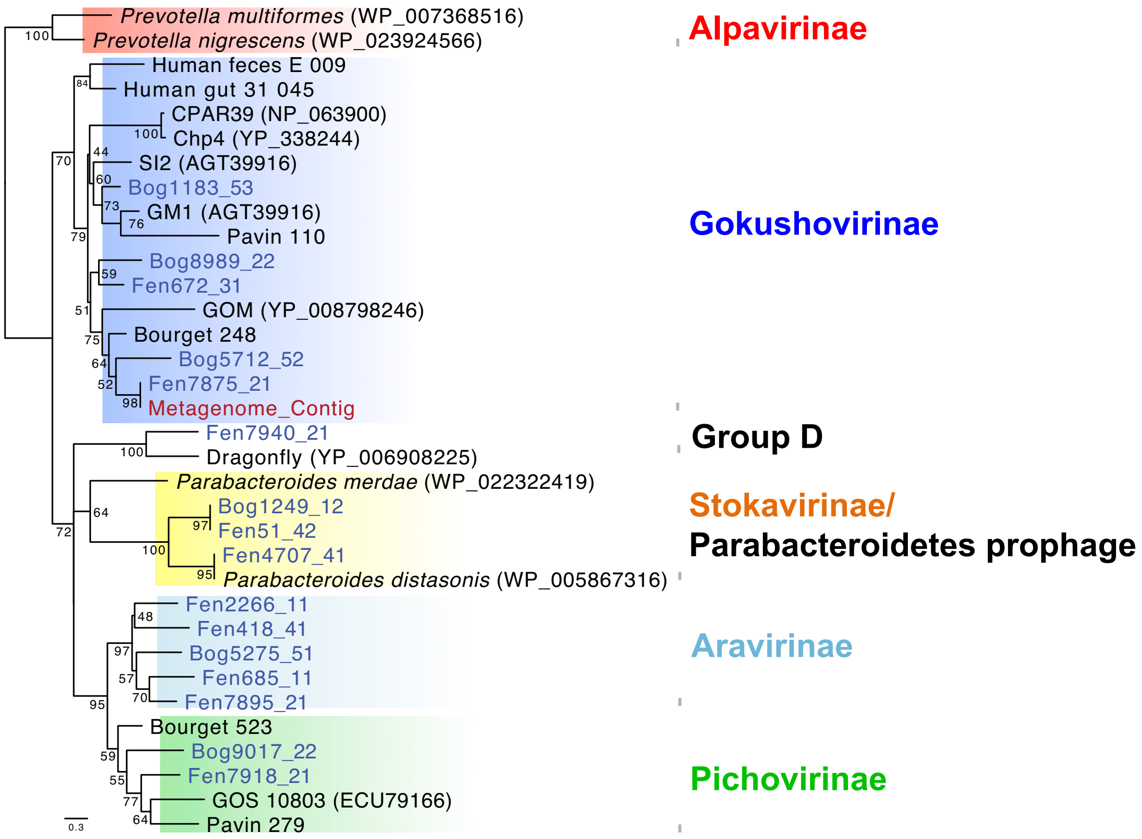

Supplementary Figure S2 Maximum likelihood phylogenetic analysis of replication proteins from *Microviridae*. A total of 144 unambiguously aligned positions from 34 sequences were used in the analysis. The replication protein from Fen7786_21 was too short to align and is not included. Bootstrap values above are indicated at the nodes. The scale bar indicates the number of substitutions per position for a unit branch length.

Supplementary Table S1 Characteristics of *Microviridae* genomes assembly

Phage Genome	Original name	Clade	Sampling date	Contig length (bp)	N° of reads_assembled (Metassembler)	Average % identity of assembled reads	Coverage (x times)	Total N° of matches from the same sample (>90% id)	N° of matches from other samples (>90% id)
Fen685_11	11_Contig685	*Aravirinae*	June 2012	4653	54	99.33	4.82	48	20
Fen2266_11	11_Contig2266	*Aravirinae*	June 2012	4366	136	99.53	22.43	98	2
Bog1249_12	12_Contig1249	*Stokavirinae*	June 2012	4453	164	99.4	15.39	150	282
Fen7786_21	21_Contig7786	*Aravirinae*	Aug. 2012	4684	129	99.4	11.46	69	196
Fen7875_21	21_Contig7875	*Gokushovirinae*	Aug. 2012	4630	411	99.31	36.93	337	0
Fen7895_21	21_Contig7895	*Aravirinae*	Aug. 2012	4424	263	99.55	24.73	238	19
Fen7918_21	21_Contig7918	*Pichovirinae*	Aug. 2012	4199	89	99.52	8.82	81	0
Fen7940_21	21_Contig7940	GroupD	Aug. 2012	4453	89	99.61	8.31	89	23
Bog8989_22	22_Contig8989	*Gokushovirinae*	Aug. 2012	4656	117	99.33	10.43	90	35
Bog9017_22	22_Contig9017	*Aravirinae*	Aug. 2012	4671	458	99.44	40.69	396	0
Fen672_31	31_Contig672	*Gokushovirinae*	Oct. 2012	4622	200	99.43	17.70	80	27
Fen418_41	41_Contig418	*Aravirinae*	Mars 2013	4949	147	99.44	12.33	103	1932
Fen4707_41	41_Contig4707	*Stokavirinae*	Mars 2013	4552	215	99.4	19.60	192	242
Fen51_42	42_Contig51	*Stokavirinae*	Mars 2013	4437	300	98.94	28.13	260	170
Bog5275_51	51_Contig5275	*Aravirinae*	Mars 2013	4815	470	99.52	40.61	442	25
Bog5712_52	52_Contig5712	*Gokushovirinae*	Mars 2013	4499	671	99.44	61.75	179	13
Bog1183_53	53_Contig1183	*Gokushovirinae*	Mars 2013	4609	1051	99.4	94.63	357	125
	Metagenome								335
average						99.41	26.99		

Supplementary Table S2 Distance matrix of whole genome phylogeny (Figure 4) based on 2052 unambiguously aligned positions.

	**Chp1**	**Chp3**	**Chp2**	**Chp4**	**CPAR39**	**phiCPG**	**SpV4**	**Bog8989_22**	**Bog1183_53**	**Fen672_31**	**MPSH06248_GM2**	**SI1**	**Bog5712_52**	**Fen7875_21**	**Metagenome**	**MPOG05594_GM1**	**SOG1**	**phiMH2K**	**SARsphi2**	**GOM**	**SI2**	**SOG2**
Chp1 (Chlamydia, 9629143)	100	59.7	59.6	60.2	59.6	59.8	49	56.1	60.4	55.5	52.6	54.8	54.6	52.9	52.9	50	53.2	48.6	48.4	51.5	55.6	54.3
Chp3 (Chlamydia, 47566140)	59.7	100	97	95.6	95	94.7	50.6	58.5	62.4	59	55.9	57.3	58.6	57.7	57.7	55.9	57.7	53.2	55	55.2	61	59.2
Chp2 (Chlamydia, 9634948)	59.6	97	100	95.6	94.6	94.8	50.5	58.3	62.4	59.3	55.8	57.6	58.7	58	58	55.9	57.7	53.9	55.1	55.4	61.1	59.6
Chp4 (Chlamydia, 77020114)	60.2	95.6	95.6	100	96.1	96.3	50.5	57.9	62.1	59	55.8	57.3	58.3	57.5	57.5	55.8	57.7	53.5	54.3	55.3	60.6	59.8
CPAR39 (Chlamydia, 9791176)	59.6	95	94.6	96.1	100	97.9	50.5	57.7	62.3	59	55.3	57.5	58.9	57.5	57.5	56	57.8	53.4	54.5	55.4	61.4	59.3
phiCPG (Chlamydia, 9632287)	59.8	94.7	94.8	96.3	97.9	100	50.5	57.7	62.6	58.9	55.8	57.5	58.7	57.7	57.7	55.8	57.8	53.2	54.3	55.3	61.4	59.1
SpV4 (Spiroplasma, 19387568)	49	50.6	50.5	50.5	50.5	50.5	100	50.5	53.7	48.2	45.9	48.2	46.8	46.3	46.3	45.7	46.1	44.3	44.1	49.8	49.2	51
**Bog8989_22**	56.1	58.5	58.3	57.9	57.7	57.7	50.5	100	62.9	59.1	55.9	56.5	57.9	57.7	57.7	51.4	56	50.5	51.7	53.5	56.3	55.2
**Bog1183_53**	60.4	62.4	62.4	62.1	62.3	62.6	53.7	62.9	100	63.6	58.4	59.6	60.1	59.3	59.3	56.1	57.4	48.2	50.3	53.6	59.5	58.1
**Fen672_31**	55.5	59	59.3	59	59	58.9	48.2	59.1	63.6	100	58.5	57.8	61.1	62.2	62.2	53.2	59.5	49.8	50.1	52.7	60.1	57
MPSH06248_GM2 (444297971)	52.6	55.9	55.8	55.8	55.3	55.8	45.9	55.9	58.4	58.5	100	57.4	58.6	57.8	57.8	49.7	56.5	48.1	51.9	51.8	55.6	54.6
SI1 (Gokushovirus, 530695349)	54.8	57.3	57.6	57.3	57.5	57.5	48.2	56.5	59.6	57.8	57.4	100	60.4	60.2	60.2	51.1	56.5	50.6	52.5	53.3	58.7	57.4
**Bog5712_52**	54.6	58.6	58.7	58.3	58.9	58.7	46.8	57.9	60.1	61.1	58.6	60.4	100	64.7	64.7	51.7	57.8	50	51.4	51.6	58.1	55.6
**Fen7875_21**	52.9	57.7	58	57.5	57.5	57.7	46.3	57.7	59.3	62.2	57.8	60.2	64.7	100	100	51.4	58.7	50	52	52	56.7	54.2
Metagenome	52.9	57.7	58	57.5	57.5	57.7	46.3	57.7	59.3	62.2	57.8	60.2	64.7	100	100	51.4	58.7	50	52	52	56.7	54.2
MPOG05594_GM1 (444297902)	50	55.9	55.9	55.8	56	55.8	45.7	51.4	56.1	53.2	49.7	51.1	51.7	51.4	51.4	100	52.7	49	48.8	50.4	53.6	52.3
SOG1 (Gokushovirus, 530695370)	53.2	57.7	57.7	57.7	57.8	57.8	46.1	56	57.4	59.5	56.5	56.5	57.8	58.7	58.7	52.7	100	52.6	53.6	55.3	58.2	57.3
phiMH2K (Bdellovibrio, 12085135)	48.6	53.2	53.9	53.5	53.4	53.2	44.3	50.5	48.2	49.8	48.1	50.6	50	50	50	49	52.6	100	53.6	51.9	53.2	53.8
SARssphi2 (313766921)	48.4	55	55.1	54.3	54.5	54.3	44.1	51.7	50.3	50.1	51.9	52.5	51.4	52	52	48.8	53.6	53.6	100	53.8	55.6	55.1
GOM Gokushovirus, 530695342)	51.5	55.2	55.4	55.3	55.4	55.3	49.8	53.5	53.6	52.7	51.8	53.3	51.6	52	52	50.4	55.3	51.9	53.8	100	58.3	60
SI2 (Gokushovirus, 530695360)	55.6	61	61.1	60.6	61.4	61.4	49.2	56.3	59.5	60.1	55.6	58.7	58.1	56.7	56.7	53.6	58.2	53.2	55.6	58.3	100	66.5
SOG2 (Gokushovirus, 530695382)	54.3	59.2	59.6	59.8	59.3	59.1	51	55.2	58.1	57	54.6	57.4	55.6	54.2	54.2	52.3	57.3	53.8	55.1	60	66.5	100

Grey shadings indicate different levels of percentage identities. White: < 50%, light grey >50% - 60%; grey > 60%; dark grey > 90%; black = 100%

Supplementary Table S3 Major capsid protein matching sequences (VP1) and general characteristics of viromes analyzed

	Sample name	Availability	Sample origin	Sample type	N° of seqs	Average size (bp)	N° of VP1 matches (Microviridae)	Reference
	**vFen June11**	Metavir id 1368	Sphagnum dominated peatland	Fen soil	90 933	415	1597	This study
	**vBog June11**	Metavir id 1369	Sphagnum dominated peatland	Bog soil	39 125	418	936	This study
	**vFen Aug11**	Metavir id 1373	Sphagnum dominated peatland	Fen soil	106 973	416	1851	This study
	**vBog Aug11**	Metavir id 1374	Sphagnum dominated peatland	Bog soil	171 231	415	931	This study
	**vFen Oct 1**	Metavir id 1375	Sphagnum dominated peatland	Fen soil	67 298	409	828	This study
	**vBog Oct11**	Metavir id 1376	Sphagnum dominated peatland	Bog soil	105 029	413	116	This study
	**vFen Mar12_A**	Metavir id 1377	Sphagnum dominated peatland	Fen soil	75 147	415	1107	This study
	**vFen Mar12_B**	Metavir id 1378	Sphagnum dominated peatland	Fen soil	62 351	416	470	This study
	**vFen Mar12_C**	Metavir id 1379	Sphagnum dominated peatland	Fen soil	78 081	416	1229	This study
	**vBog Mar12_A**	Metavir id 1380	Sphagnum dominated peatland	Bog soil	114 473	416	1429	This study
	**vBog Mar12_B**	Metavir id 1382	Sphagnum dominated peatland	Bog soil	122 143	414	5301	This study
	**vBog Mar12_C**	Metavir id 1370	Sphagnum dominated peatland	Bog soil	65 424	415	1192	This study
1	Lake Pavin	Metavir (id: 6)	Lake Pavin - France	Freshwater	649 290	412	1601	(Roux et al., 2012)
2	Lake Bourget	Metavir (id: 7)	Lake Bourget - France	Freshwater	593 084	433	36879	(Roux et al., 2012)
3	Airborn Forest	Metavir id 1050	Central Region Korea	Air	23 316	544	85	(Whon et al., 2012)
4	Airborn Industrial Komplex	Metavir id 1051	Korea	Air	20 578	539	403	(Whon et al., 2012)
5	Airborn Rainwater	Metavir id 1052	Seoul city - Korea	Air	18 829	542	24	(Whon et al., 2012)
6	Airborn Residential	Metavir id 1053	Seoul city - Korea	Air	24 721	542	71	(Whon et al., 2012)
7	Sediment-Ogasawara	Metavir id 164	Ogasawara Trench - Japan	Pelagic sediment	46 458	336	3058	(Yoshida et al., 2013)
8	Sediment-Mariana	Metavir id 165	Mariana Trench - Japan	Pelagic sediment	49 584	279	173	(Yoshida et al., 2013)
9	Sediment-Shimokita	Metavir id 166	Shimokita Peninsula- Japan	Pelagic sediment	76 498	365	5598	(Yoshida et al., 2013)
10	OMZ st3 viral 10m	Metavir id 897	Chile	Marine OMZ	128 441	246	380	(Cassman et al., 2012)
11	OMZ st3 viral 200m	Metavir id 898	Chile	Marine OMZ	96 706	164	3	(Cassman et al., 2012)
12	OMZ st3 viral 90m	Metavir id 992	Chile	Marine OMZ	361 488	253	34	(Cassman et al., 2012)
13	OMZ st5 viral 200m	Metavir id 993	Chile	Marine OMZ	163 531	242	4	(Cassman et al., 2012)
14	OMZ st5 viral 55m	Metavir id 899	Chile	Marine OMZ	226 628	246	6	(Cassman et al., 2012)
15	OMZ st5 viral 90m	Metavir id 901	Chile	Marine OMZ	300 835	252	9	(Cassman et al., 2012)
16	Human feces A	Metavir id 196	Healthy human	Human feces	113 054	431	22 918	(Kim et al., 2011)
17	Human feces B	Metavir id 293	Healthy human	Human feces	109 569	435	15 619	(Kim et al., 2011)
18	Human feces C	Metavir id 298	Healthy human	Human feces	68 391	437	5 275	(Kim et al., 2011)
19	Human feces D	Metavir id 299	Healthy human	Human feces	115 121	433	20 706	(Kim et al., 2011)
20	Human feces E	Metavir id 300	Healthy human	Human feces	98 511	417	22 458	(Kim et al., 2011)
21	Indian ocean GS108	Metavir id 1478	Coccos Keeling - Australia	Marine plancton (1.8m)	320 104	391	1	(Williamson et al., 2012)
22	Indian ocean GS112	Metavir id 1477	Indian ocean - Australia	Marine plancton (8m)	494 832	361	0	(Williamson et al., 2012)
23	Indian ocean GS117	Metavir id 1479	Saint-Anne Island -Seychelles	Marine plancton (1.8m)	480 375	404	0	(Williamson et al., 2012)
24	Indian ocean GS122	Metavir id 1480	Madagaskar-South Africa	Marine plancton (1.9m)	341 386	350	0	(Williamson et al., 2012)
25	Human salivary Sub1 D1	Metavir id 711	USA	Human salivary	63476	443	8	(Pride et al., 2012)
26	Human salivary Sub1 D30	Metavir id 712	USA	Human salivary	86362	439	0	(Pride et al., 2012)
27	Human salivary Sub2 D30	Metavir id 1102	USA	Human salivary	119 621	406	1 671	(Pride et al., 2012)
28	Human salivary Sub3 D30	Metavir id 1147	USA	Human salivary	103744	287	10	(Pride et al., 2012)
29	Human salivary Sub5 D1	Metavir id 1148	USA	Human salivary	604 957	306	110 355	(Pride et al., 2012)
30	Saanich Inlet (10-200m)	CAMERA	Saanich Inlet - USA	Seawater	96 950	512	269	(Labonté and Suttle, 2013)
31	Gulf of Mexico GOM	CAMERA	Gulf of Mexico - USA	Seawater	87 274	483	149	(Labonté and Suttle, 2013)
32	Coastal water SOG	CAMERA	British Columbia Strait - USA	Seawater	95 402	541	1 184	(Labonté and Suttle, 2013)
33	V1 Typhoon freshwater	SRR371573	Taiwan	Freshwater reservoir	110 339	245	935	(Tseng et al., 2013)
34	V2 Typhoon freshwater	SRR371574	Taiwan	Freshwater reservoir	93 171	247	520	(Tseng et al., 2013)
35	V3 Typhoon freshwater	SRR648311	Taiwan	Freshwater reservoir	75 645	250	37	(Tseng et al., 2013)
36	V4 Typhoon freshwater	SRR648312	Taiwan	Freshwater reservoir	67 123	254	591	(Tseng et al., 2013)
37	V5 Typhoon freshwater	SRR648313	Taiwan	Freshwater reservoir	61 773	254	653	(Tseng et al., 2013)
38	V6 Typhoon freshwater	SRR648314	Taiwan	Freshwater reservoir	59 007	263	11	(Tseng et al., 2013)
39	Reclaimed water Effluent DNA	Metavir id 1351	USA ((FL)	Freshwater	262 097	245	54	(Rosario et al., 2009)
40	Reclaimed water Effluent RNA	Metavir id 1352	USA ((FL)	Freshwater	247 589	227	0	(Rosario et al., 2009)
41	Reclaimed water NurseryDNA	Metavir id 1353	USA ((FL)	Freshwater	283 753	243	3197	(Rosario et al., 2009)
42	Reclaimed water Nursery RNA	Metavir id 1354	USA ((FL)	Freshwater	295 823	236	0	(Rosario et al., 2009)
43	Reclaimed water Park DNA	Metavir id 1355	USA ((FL)	Freshwater	202 436	105	1	(Rosario et al., 2009)
44	Potable Water	Metavir id 1356	USA ((FL)	Freshwater	240 259	226	59	(Rosario et al., 2009)
45	Antarctic Lake Spring	Metavir id 10	Livingston Island, Antarctica	Freshwater	41 322	237	277	(López-Bueno et al., 2009)
46	Antarctic Lake Summer	Metavir id 11	Livingston Island, Antarctica	Freshwater	38 475	221	34	(López-Bueno et al., 2009)
47	Coral_A1	Metavir id 850	US Virgin Islands (Brewers_Bay)	Coral	5 677	385	2	(Soffer et al., 2014)
48	Coral A10	Metavir id 879	US Virgin Islands (Brewers_Bay)	Coral	15 939	404	1352	(Soffer et al., 2014)
49	Coral A2	Metavir id 851	US Virgin Islands (Brewers_Bay)	Coral	3 033	386	303	(Soffer et al., 2014)
50	Coral A3	Metavir id 852	US Virgin Islands (Brewers_Bay)	Coral	4 068	399	128	(Soffer et al., 2014)
51	Coral A5	Metavir id 853	US Virgin Islands (Brewers_Bay)	Coral	24 953	405	5485	(Soffer et al., 2014)
52	Coral A6	Metavir id 854	US Virgin Islands (Brewers_Bay)	Coral	11 924	381	157	(Soffer et al., 2014)
53	Coral A9	Metavir id 855	US Virgin Islands (Brewers_Bay)	Coral	12 942	380	0	(Soffer et al., 2014)
54	Coral D1	Metavir id 880	US Virgin Islands (Brewers_Bay)	Coral	60 629	398	5607	(Soffer et al., 2014)
55	Coral D10	Metavir id 886	US Virgin Islands (Brewers_Bay)	Coral	12 313	395	4073	(Soffer et al., 2014)
56	Coral D2	Metavir id 881	US Virgin Islands (Brewers_Bay)	Coral	14 905	393	214	(Soffer et al., 2014)
57	Coral D3	Metavir id 882	US Virgin Islands (Brewers_Bay)	Coral	25 484	393	3849	(Soffer et al., 2014)
58	Coral D5	Metavir id 883	US Virgin Islands (Brewers_Bay)	Coral	20 186	392	31	(Soffer et al., 2014)
59	Coral D6	Metavir id 884	US Virgin Islands (Brewers_Bay)	Coral	27065	381	1597	(Soffer et al., 2014)
60	Coral D9	Metavir id 885	US Virgin Islands (Brewers_Bay)	Coral	27065	380	1597	(Soffer et al., 2014)
61	Coral H10	Metavir id 892	US Virgin Islands (Brewers_Bay)	Coral	698	400	140	(Soffer et al., 2014)
62	Coral H2	Metavir id 887	US Virgin Islands (Brewers_Bay)	Coral	17786	392	15	(Soffer et al., 2014)
63	Coral H3	Metavir id 888	US Virgin Islands (Brewers_Bay)	Coral	2336	407	0	(Soffer et al., 2014)
64	Coral H5	Metavir id 889	US Virgin Islands (Brewers_Bay)	Coral	9409	392	0	(Soffer et al., 2014)
65	Coral H6	Metavir id 890	US Virgin Islands (Brewers_Bay)	Coral	25037	381	5	(Soffer et al., 2014)
66	Coral_sur. Water d	Metavir id 895	US Virgin Islands (Brewers_Bay)	Coral	22685	407	1330	(Soffer et al., 2014)
67	Coral sur. Water H	Metavir id 896	US Virgin Islands (Brewers_Bay)	Coral	17405	392	1405	(Soffer et al., 2014)
68	Coral Ub1	Metavir id 893	US Virgin Islands (Brewers_Bay)	Coral	1608	381	0	(Soffer et al., 2014)
69	Coral Ub2	Metavir id 894	US Virgin Islands (Brewers_Bay)	Coral	9993	407	1495	(Soffer et al., 2014)

References to Supplementary Table S3

Cassman, N., Prieto-Davó, A., Walsh, K., Silva, G. G. Z., Angly, F., Akhter, S., Barott, K., Busch, J., McDole, T., Haggerty, J. M., et al. (2012). Oxygen minimum zones harbour novel viral communities with low diversity. *Environ Microbiol* 14, 3043–3065. doi:10.1111/j.1462-2920.2012.02891.x.

Kim, M.-S., Park, E.-J., Roh, S. W., and Bae, J.-W. (2011). Diversity and abundance of single-stranded DNA viruses in human feces. *Applied and Environmental Microbiology* 77, 8062–8070. doi:10.1128/AEM.06331-11.

Labonté, J. M., and Suttle, C. A. (2013). Previously unknown and highly divergent ssDNA viruses populate the oceans. *ISME J* 7, 2169–2177. doi:10.1038/ismej.2013.110.

López-Bueno, A., Tamames, J., Velázquez, D., Moya, A., Quesada, A., and Alcamí, A. (2009). High diversity of the viral community from an Antarctic lake. *Science* 326, 858–861. doi:10.1126/science.1179287.

Pride, D. T., Salzman, J., Haynes, M., Rohwer, F., Davis-Long, C., White, R. A., Loomer, P., Armitage, G. C., and Relman, D. A. (2012). Evidence of a robust resident bacteriophage population revealed through analysis of the human salivary virome. *ISME J* 6, 915–926. doi:10.1038/ismej.2011.169.

Rosario, K., Nilsson, C., Lim, Y. W., Ruan, Y., and Breitbart, M. (2009). Metagenomic analysis of viruses in reclaimed water. *Environ Microbiol* 11, 2806–2820. doi:10.1111/j.1462-2920.2009.01964.x.

Roux, S., Enault, F., Robin, A., Ravet, V., Personnic, S., Theil, S., Colombet, J., Sime-Ngando, T., and Debroas, D. (2012). Assessing the diversity and specificity of two freshwater viral communities through metagenomics. *PLoS ONE* 7, e33641. doi:10.1371/journal.pone.0033641.

Soffer, N., Brandt, M. E., Correa, A. M. S., Smith, T. B., and Thurber, R. V. (2014). Potential role of viruses in white plague coral disease. *ISME J* 8, 271–283. doi:10.1038/ismej.2013.137.

Tseng, C.-H., Chiang, P.-W., Shiah, F.-K., Chen, Y.-L., Liou, J.-R., Hsu, T.-C., Maheswararajah, S., Saeed, I., Halgamuge, S., and Tang, S.-L. (2013). Microbial and viral metagenomes of a subtropical freshwater reservoir subject to climatic disturbances. *ISME J* 7, 2374–2386. doi:10.1038/ismej.2013.118.

Whon, T. W., Kim, M.-S., Roh, S. W., Shin, N.-R., Lee, H.-W., and Bae, J.-W. (2012). Metagenomic characterization of airborne viral DNA diversity in the near-surface atmosphere. *J. Virol.* 86, 8221–8231. doi:10.1128/JVI.00293-12.

Williamson, S. J., Allen, L. Z., Lorenzi, H. A., Fadrosh, D. W., Brami, D., Thiagarajan, M., McCrow, J. P., Tovchigrechko, A., Yooseph, S., and Venter, J. C. (2012). Metagenomic exploration of viruses throughout the Indian Ocean. *PLoS ONE* 7, e42047. doi:10.1371/journal.pone.0042047.

Yoshida, M., Takaki, Y., Eitoku, M., Nunoura, T., and Takai, K. (2013). Metagenomic analysis of viral communities in (hado)pelagic sediments. *PLoS ONE* 8, e57271. doi:10.1371/journal.pone.0057271.
